# *Leishmania infantum* and Human Visceral Leishmaniasis, Argentina

**DOI:** 10.3201/eid1802.110924

**Published:** 2012-02

**Authors:** Alejandra Barrio, Cecilia M. Parodi, Fabricio Locatelli, María C. Mora, Miguel A. Basombrío, Masataka Korenaga, Yoshihisa Hashiguchi, María F. García Bustos, Alberto Gentile, Jorge D. Marco

**Affiliations:** Universidad Nacional de Salta, Salta, Argentina (A. Barrio, C.M. Parodi, M.C. Mora, M.A. Basombrío, M.F. García Bustos, J.D. Marco);; Kochi Medical School, Kochi, Japan (F. Locatelli, M. Korenaga, Y. Hashiguchi);; Ministerio de Salud Pública-Provincia de Salta, Salta (A. Gentile)

**Keywords:** visceral leishmaniasis, cutaneous leishmaniasis, Leishmania infantum, parasites, molecular characterization, cytochrome b gene sequence, PCR, Salta, Argentina

**To the Editor**: In Argentina, 14 autochthonous human cases of visceral leishmaniasis (VL) were reported during 1925–1989. These cases occurred in different localities in Salta, Jujuy, Santiago del Estero, and Chaco Provinces of northwestern Argentina (Figure A1), where cutaneous leishmaniasis (CL) caused principally by *Leishmania* (*Viannia*) *braziliensis* is endemic.

It had been postulated that scattered/sporadic VL cases could be caused by visceralization of dermatrophic *Leishmania* spp. because of 1) absence of already recognized *L.* (*Leishmania*) *infantum* vector species; 2) geographic overlap with the region where CL is endemic; 3) simultaneous symptoms of CL; or 4) lack of detailed parasitic characterization at the molecular level for cases of suspected VL (*1*). However, during recent decades, urban outbreaks of VL have spread to southern regions of South America (Mato Grosso do Sul, Brazil, and Asunción, Paraguay) near the northern border with Argentina.

In May 2006, an autochthonous human case of VL was reported in Posadas (northeastern Argentina); it was associated with the canine visceral form of the disease. In addition, the presence of *Lutzomyia longipalpis* sandflies was also reported (*2*). Currently, 58 human VL cases have been reported in Posadas (*3*), and >7,000 infected dogs, *Lu. longipalpis* sandflies, and canine VL have been found 350 km south of Posadas (*4*).

During 2007–2008, new VL cases in 4 children and 7 dogs were reported clustering in time and space in La Banda-Santiago del Estero in the dry Chaco region of Argentina. This focus showed a different pattern from that found in the only urban outbreak of VL reported (nearly the same number of cases in humans and dogs, and the suspected vector was *Lu. migonei* sandflies instead of *Lu. longipalpis* sandflies) (*5*).

We report a case of autochthonous human VL in Salta Argentina that was caused by *L*. (*L.*) *infantum*. This parasite was characterized by cytochrome *b* (*cytb*) gene sequencing. Sequencing of this gene has been validated for precise characterization of *Leishmania* spp (*6**,**7*).

On September 9, 2009, a 44-year-old man from Salta, Argentina (Figure A1), was admitted to the Infectious Disease Service at Hospital Señor del Milagro in Salta. The patient had fever, weight loss, dyspepsia, and splenomegaly that evolved over 3 weeks. Physical examination showed cutaneous and mucosal paleness**.** His general condition was feverish and rapidly deteriorating.

Laboratory tests at the time of final diagnosis showed anemia, leukocytopenia, thrombocytopenia, and increased levels of lactate dehydrogenase. Results of urinalysis and coproculture were negative for parasites. Electrophoresis of serum proteins showed increased levels of gamma globulins. The differential diagnosis was negative for malaria, mycosis, autoimmune hepatitis, and lymphoma. A bone marrow smear showed abundant amastigotes by Giemsa staining (Figure, panel A). The patient was treated with liposomal amphotericin B, 3 mg/day for 7 days, and recovered (*8*).

After a comprehensive interview, we verified that this patient had not been in the VL-endemic area in Argentina. However, he had worked (deforestation activities) during January–February 2009 on a farm in Finca Las Maravillas (22º3′29.30″S, 63º14′28.17″W), where he had been bitten by phlebotomines and acquired the disease. This farm was situated in the dry Chaco region near the border with Bolivia and Paraguay (zones with VL) (*9*), a region with intensive deforestation and agricultural activities.

For species identification, DNA was extracted from a bone marrow aspirate and peripheral blood. We amplified by nested PCR and sequenced the *cytb* gene (Figure, panel B) (*6*). The aligned 817-bp sequence obtained showed 100% homology with the *cytb* gene of the MHOM/TN/80/IPT1 *L.* (*L.*) *infantum* World Health Organization reference strain (Tunisian strain) and 99.3% homology with the MHOM/BR/74/PP75 *L.* (*L.*) *chagasi* strain (Brazilian strain) (*7*).

*L.* (*L.*) *infantum* was identified as the causative agent of this VL case in Salta, Argentina, where VL cases had not been seen for 50 years. Our findings indicate that this case was not caused by visceralization or a dermatropic *Leishmania* spp. We suggest that the scattered pattern of VL incidence in the dry Chaco region is caused by an enzootic cycle with accidental human transmission (*5*).

There are no reports of *Lu. longipalpis* sandflies in the study area or surrounding areas (*10*). Nevertheless, studies on natural infections of vector sandflies and reservoir-host animals (especially dogs) are needed. Therefore, the search for naturally infected sandflies and reservoirs of this infection should be intensified. Epidemiologic surveys of dogs are needed to identify spread of VL foci in areas of deforestation. Deforestation could alter vector and reservoir range and parasite density in the enzootic cycle and increase human exposure to infected vectors.

**Figure:**
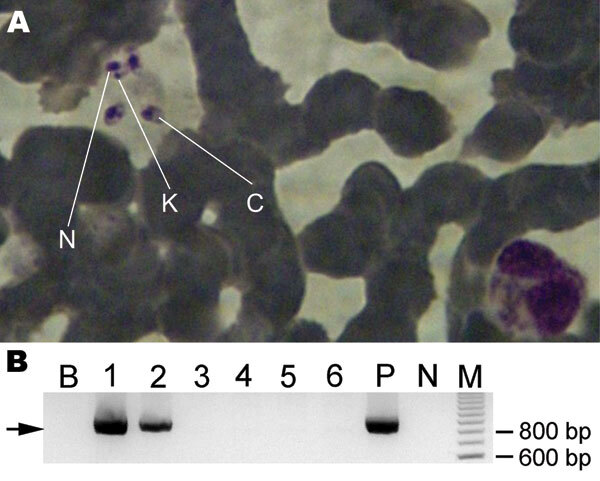
Case of autochthonous human visceral leishmaniasis in a 44-year-old man, identified by parasitologic diagnosis and molecular detection of the causative species, Salta, Argentina. A) *Leishmania* amastigotes in a bone marrow smear. N, nucleus; K, kinetoplast; C, cytoplasm (Giemsa stained, original magnification ×1,000). B) Amplification by nested PCR of cytochrome *b* gene of *Leishmania infantum*. Arrow indicates amplified fragment of ≈850 bp. Lane B, blank control; lanes 1 and 2, patient bone marrow aspirate samples; lanes 3–6, samples from *Leishmania* spp.–negative persons; lane P, positive control; lane N, negative control; lane M, 100-bp molecular mass marker.

## Figures and Tables

**Figure A1:**
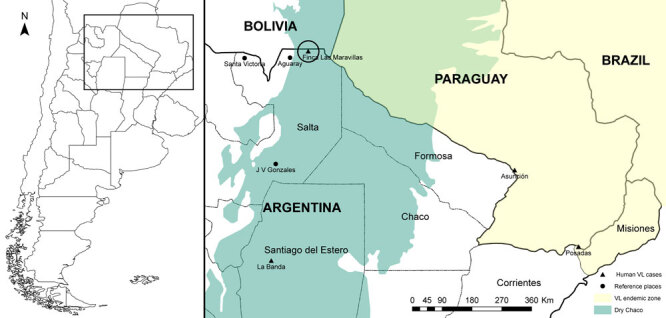
Incidence of visceral leishmaniasis (VL) in northern Argentina. Map at left indicates location of study area (right). Circle indicates location of farm where the patient (44-year-old man) in this study worked. Light green area indicates dry Chaco region, where VL is endemic and human cases have been found.
